# *Bacteroides fragilis* metabolises exopolysaccharides produced by bifidobacteria

**DOI:** 10.1186/s12866-016-0773-9

**Published:** 2016-07-15

**Authors:** David Rios-Covian, Isabel Cuesta, Jorge R. Alvarez-Buylla, Patricia Ruas-Madiedo, Miguel Gueimonde, Clara G. de los Reyes-Gavilán

**Affiliations:** Probiotics and Prebiotics Group, Department of Microbiology and Biochemistry of Dairy Products, Instituto de Productos Lácteos de Asturias, Consejo Superior de Investigaciones Científicas (IPLA-CSIC), Villaviciosa, Asturias Spain; Scientific and Technical Facilities, Instituto de Productos Lácteos de Asturias, Consejo Superior de Investigaciones Científicas (IPLA-CSIC), Villaviciosa, Asturias Spain

**Keywords:** Bacteroides fragilis, Exopolysaccharides, Bifidobacterium, MALLS, Heteropolysaccharides

## Abstract

**Background:**

*Bacteroides fragilis* is the most frequent species at the human intestinal mucosal surface, it contributes to the maturation of the immune system although is also considered as an opportunistic pathogen. Some *Bifidobacterium* strains produce exopolysaccharides (EPS), complex carbohydrate polymers that promote changes in the metabolism of *B. fragilis* when this microorganism grows in their presence. To demonstrate that *B. fragilis* can use EPS from bifidobacteria as fermentable substrates, purified EPS fractions from two strains, *Bifidobacterium longum* E44 and *Bifidobacterium animalis* subsp. *lactis* R1, were added as the sole carbon source in cultures of *B. fragilis* DSMZ 2151 in a minimal medium. Bacterial counts were determined during incubation and the evolution of organic acids, short chain fatty acids (SCFA) and evolution of EPS fractions was analysed by chromatography.

**Results:**

Growth of *B. fragilis* at early stages of incubation was slower in EPS than with glucose, microbial levels remaining higher in EPS at prolonged incubation times. A shift in metabolite production by *B. fragilis* occurred from early to late stages of growth, leading to the increase in the production of propionate and acetate whereas decrease lactate formation. The amount of the two peaks with different molar mass of the EPS E44 clearly decreased along incubation whereas a consumption of the polymer R1 was not so evident.

**Conclusions:**

This report demonstrates that *B. fragilis* can consume some EPS from bifidobacteria, with a concomitant release of SCFA and organic acids, suggesting a role for these biopolymers in bacteria-bacteria cross-talk within the intestine.

**Electronic supplementary material:**

The online version of this article (doi:10.1186/s12866-016-0773-9) contains supplementary material, which is available to authorized users.

## Background

The colonic microbiota is a complex community whose metabolic activity influences our health and nutritional status through diverse pathways [1]. Microbiota of adult healthy people is dominated by the phyla Firmicutes and Bacteroidetes, even though the composition at the species level is highly variable among individuals [2, 3]. Members of this microbiota are able to degrade complex polysaccharides and, therefore, genes involved in the degradation and consumption of these compounds are widespread among the genomes of microbiota-resident species [4, 5]. In this respect, the genus *Bacteroides* has the ability to use a wide range of carbohydrates, this ability varying as depending on the species considered [6]. *Bacteroides* is an anaerobic, bile-resistant, non-spore forming, and Gram negative rod [7] that accounts for up to 20–50 % of the total microbiota in most individuals [8]. *Bacteroides fragilis* is the most frequent species at the mucosal surface [9] and can contribute to the development and maturation of the host immune system [10]. This species has an extraordinarily good adaptability to environmental changes due to its capacity to regulate the cell surface [11]. Consequently *B. fragilis* is the clinical isolate most frequently found in systemic infections, this microorganism being then considered as an opportunistic pathogen [7].

Exopolysaccharides (EPS) are complex carbohydrates located outside the cell; some *Bifidobacterium* strains, as well as many other microorganisms, are able to produce these polymers [12]. The synthesis of these compounds in the intestine has not been demonstrated yet. However, it has been proven *in vitro* that the presence of bile, which is released to the small intestine during digestion, stimulates the production of EPS by bifidobacteria [13]. EPS could be constituted either by a unique type of monomer, named as homopolysaccharides (HoPS), or by more than one monosaccharide type, then known as heteropolysaccharides (HePS). All bifidobacterial EPS characterized until present are HePS [14].

Differential growth of members from the genus *Bacteroides* in the presence of EPS isolated from *Lactobacillus* and *Bifidobacterium* strains has been previously reported [15, 16]. Particularly, two EPS fractions isolated from *Bifidobacterium longum* E44 and *Bifidobacterium animalis* subsp. *lactis* R1, have shown the capacity to act as fermentable substrates by the intestinal microbiota, thus promoting the increase of *Bacteroides* populations in faecal cultures [15, 17]. Specific changes have been demonstrated as well in the metabolism of *B. fragilis* when grown in an undefined and complex medium in the presence of EPS E44 and R1 as compared with cultures in glucose [18]. Although these studies suggest that *Bacteroides* can use bifidobacterial EPS as fermentable substrates, this had not yet been effectively proven. In the present work, we assessed this question by testing the ability of *B. fragilis* DSMZ 2151 to growth in a minimal medium at the expenses of EPS from bifidobacteria when they are the sole carbon and energy source available.

## Methods

### Bacterial strains and culture media

Frozen stocks of *B. fragilis* DSMZ 2151 (DSMZ bacterial pure collection, Braunschewig, Germany) were reactivated in Gifu anaerobic medium (GAM) broth (Nissui Pharmaceutical Co., Tokyo, Japan) supplemented with 0.25 % (w/v) L-cysteine (Sigma Chemical co., St. Louis, MO, USA) (named GAMc) and incubated overnight at 37 °C in an anaerobic cabinet (Mac 100; Don Whitley Scientific, West Yorkshire, UK) under a 10 % H_2_, 10 % CO_2_, and 80 % N_2_ atmosphere. pH free batch cultures of *B. fragilis* were performed in a modified minimal medium (MM) previously used in *B. fragilis* metabolism studies [19]. Briefly, the medium contained per litre: (NH_4_)_2_SO_4_, 1 g; KH_2_PO_4_, 0.9 g; NaCl, 0.9 g; CaCl_2_ · 2H_2_0, 26.5 mg; MgCl · 6H_2_0, 20 mg; MnCl_2_ · 4H_2_0, 10 mg; FeSO_4_ · 7H_2_0, 4 mg; CoCl_2_ · 6H_2_0, 1 mg; resazurin, 1 mg; vitamin B_12_, 10 μg; vitamin K_1_, 2 mg; and haemin, 4 mg. MM was supplemented with 0.5 % (w/v) of glucose, EPS E44 or EPS R1 and the final pH of the medium ranged between 7.6 and 7.9. MM was inoculated with a 1 % (v/v) overnight culture of *B. fragilis* in GAMc in a final volume of 10 mL. A culture of *B. fragilis* inoculated in MM without carbon source added was used as a negative control. Potential changes over time in the characteristics of the EPS fractions during incubation not due to the microbial action were monitored in uninoculated MM added with EPS. Bacterial growth was monitored by counting in agar-GAMc plates at 0, 24, 48, 72 and 144 h of incubation. Experiments were run in triplicate using the same inoculum in all conditions.

### EPS isolation

The EPS fractions were obtained from the strains *B. animalis* subsp*. lactis* IPLA R1 [20], a dairy origin strain, and *B. longum* IPLA E44 [21], a faecal isolate from a healthy adult faeces. Cellular biomass was harvested with ultrapure water from agar-MRS (Biokar, Allone, France) plates with 0.25 % (w/v) L-cysteine (Sigma) and incubated for 3 days at 37 °C under anaerobic conditions [22]. In brief, EPS was separated from the cellular biomass by gently stirring overnight with one volume of 2 M NaOH at room temperature. Then, cells were removed by centrifugation and EPS were precipitated from the supernatant with two volumes of absolute cold ethanol for 48 h at 4̊ C. After centrifugation at 10,000 x *g* for 30 min at 4̊ C, the EPS fraction was resuspended in ultrapure water and dialyzed against water during 3 days in 12- to 14-kDa molecular weight cut off dialysis tubes (Sigma). The protein content of the polymers was determined by the BCA protein assay kit (Pierce, Rockford, IL, USA) following the manufacturer’s instructions. Finally, EPS fractions were freeze-dried. The EPS E44 and R1 fractions contained 2.25 % and 1.99 % protein, respectively.

To check the purity of the EPS and test for the eventual presence of bacterial glycogen, EPS E44 and R1 fractions and glycogen (Roche, Switzerland) were digested with α-amylase (Sigma, USA) and/or pullulanase (Sigma, USA) and degradation profiles were compared. Digested samples were run in a TLC gel as described by Koropatkin and Smith [23]. In brief, each reaction mixture contained 1 mg/mL of substrate (EPS R1, EPS E44 or commercial glycogen) and 22 μg/mL of one or both enzymes in a 20 mM HEPES buffer. After 4 h of digestion at 37 °C, 6 μl of each sample reaction were placed and dried in a TLC Silica Gel 60 (Merk, Germany). Gels were transferred to a solvent chamber with a 3:1:1 mixture of isopropanol: ethylacetate: water, run for 3 h and revealed by irrigating a 5 % sulfuric dilution in ethanol and were dried at 120 C° for 10 min. (Additional file 1: Figure S1).

### Metabolite analysis and EPS molar mass distribution and quantification

Organic acids (lactic, succinic and formic) formed during incubation were analysed by HPLC. Cell-free supernatants from cultures were filtered (0.2 μm) and quantified using an Alliance 2695 module injector, a PDA 966 photodiode array detector, a 2414 differential refractometer detector and the Empower software (Walters, Mildford, MA), following the chromatographic conditions described previously [22]. The weight average molar mass (M_w_) distribution of EPS fractions was determined in the same equipment by size-exclusion chromatography (SEC) using two different columns placed in series, TSK-Gel G3000 PW_xL_ and TSK-Gel G5000 PW_xL_ (Supelco-Sigma) and the multiangle laser light scattering (MALLS) detector DawnHeleos II (Wyatt Europe GmbM, Dembach, Germany) as described previously [22]. The EPS peaks were detected and quantified with the refractive index detector, using standards of dextran (Fluka-Sigma) of different molar masses; the presence of proteins was monitored through a PDA detector set at 220 nm [17]. Short chain fatty acids (SCFA; acetic and propionic) were quantified in the supernatants by Gas Chromatography (GC) using a system composed of a 6890 N gas chromatograph (Agilent Technologies Inc., Palo Alto, CA, USA) connected with a FID detector (Agilent) and a mass spectrometry (MS) 5973 N detector (Agilent) as described previously [15]. Concentrations were expressed in millimolar (mM).

### Statistical analysis

One way ANOVA statistical tests was run to compare the evolution of the different parameters analysed along time or among cultures with the different carbon and energy sources by means of the IBM SPSS software, version 22.00 (IBM, Armonk, New York, USA). SNK post-hoc test was used when required.

## Results

The digestion pattern of EPS fractions E44 and R1 with the enzymes α-amylase (endohydrolysis of 1,4- α-D-glucosidic linkages) and pullulanase (endohydrolysis of 1,6-α-D-glucosidic linkages), were clearly different from that of glycogen (Additional file 1: Figure S1). This indicates that the release of intracellular glycogen during the process of obtaining the EPS fractions is negligible. Therefore, the results obtained through our work should be related with EPS polymers.

The metabolite production and growth pattern of *B. fragilis* DSMZ 2151 was dependent on the carbon source present in the culture medium. The final pH attained with glucose (6.52 ± 0.16) was significantly lower than with EPS E44, EPS R1, and without carbon source (*p* < 0.05) (7.62 ± 0.02, 7.66 ± 0.01 and 7.73 ± 0.05 respectively). The pH values remained unchanged in MM without carbon source added whereas bacterial counts increased 1.3 log units from time 0 (7.02 ± 0.08 log ufc mL^−1^) to 24 h of incubation in such conditions, probably due to the metabolic inertia of the inoculum (Table 1). At 24 h of incubation microbial counts reached were 1.22 log ufc mL^−1^ higher in glucose and 0.6–0.8 greater in EPS than in the control MM medium without any carbon source added (*p* < 0.05) (Table 1). From that point on, the time course of *B. fragilis* growth was different depending on the carbon source, and microbial population levels remained generally higher in EPS than in glucose at late stages of growth (from 48 h of incubation) (Table 1). Thus, microbial counts in glucose continuously decreased from 24 h to the end of incubation (*p* < 0.05). In contrast, levels of *B. fragilis* with both EPS remained largely unchanged from 24 to 72 h. From that time population levels decreased with both polymers, this decrease being much more pronounced with EPS R1 than with EPS E44.

**Table 1:** Growth and metabolic parameters of *B. fragilis* grown in glucose, EPS or without carbon source

Carbon source	Time (h)	Acetic acid	Propionic acid	Lactic acid	Succinic acid	Formic acid	Total metabolites*	Acetic/Propionic	Propionic/Succinic	Acetic/Lactic	Bacterial counts^+^
Glucose	24	13.00 ± 0.41^D^	4.35 ± 0.63^C^	14.81 ± 0.27^B^	3.46 ± 0.19^a B^	7.33 ± 0.15^D^	42.95 ± 1.40^a C^	3.02 ± 0.35^C^	1.25 ± 0.12^A^	0.88 ± 0.01^A^	9.58 ± 0.07^d C^
	48	13.28 ± 0.74^C^	4.69 ± 0.21^C^	17.11 ± 1.26^B^	3.75 ± 0.27^a B^	6.86 ± 0.38^D^	45.73 ± 1.22^ab D^	2.83 ± 0.08^D^	1.25 ± 0.13^A^	0.78 ± 0.07^A^	8.47 ± 0.16^c B^
	72	13.80 ± 0.53^C^	5.04 ± 0.47^D^	16.15 ± 1.01^B^	4.37 ± 0.18^b C^	7.59 ± 0.12^C^	47.11 ± 0.41^b C^	2.75 ± 0.18^D^	1.15 ± 0.09^A^	0.86 ± 0.09	7.76 ± 0.06^b A^
	144	13.66 ± 0.26^D^	4.96 ± 0.32^D^	15.27 ± 0.87^B^	4.56 ± 0.52^b B^	7.26 ± 0.45^C^	46.02 ± 1.96^ab D^	2.76 ± 0.15^C^	1.09 ± 0.06^A^	0.90 ± 0.05^A^	7.46 ± 0.17^a B^
EPS E44	24	1.24 ± 0.06^b B^	0.78 ± 0.08^b A,B^	0.45 ± 0.07^b A^	0.12 ± 0.02^A^	0.43 ± 0.04^a B^	3.01 ± 0.11^a B^	1.60 ± 0.10^a B^	6.78 ± 0.27^D^	2.83 ± 0.53^a A^	9.17 ± 0.12^c B^
	48	0.93 ± 0.08^a A,B^	0.39 ± 0.08^a A^	0.95 ± 0.05^c A^	-	1.27 ± 0.24^b C^	3.56 ± 0.22^b C^	2.44 ± 0.30^b C^	ND	0.98 ± 0.12^a A^	8.91 ± 0.02^b C^
	72	1.89 ± 0.10^c B^	0.95 ± 0.13^b B^	0.77 ± 0.08^c A^	-	1.67 ± 0.46^b B^	5.31 ± 0.34^c B^	2.01 ± 0.19^ab C^	ND	2.48 ± 0.38^a^	9.05 ± 0.05^bc B^
	144	5.53 ± 0.08^d C^	2.75 ± 0.13^c C^	0.26 ± 0.15^a A^	0.13 ± 0.05^A^	1.40 ± 0.31^b B^	10.07 ± 0.39^d C^	2.01 ± 0.12^ab B^	25.22 ± 11.55^B^	26.24 ± 12.29^b A^	8.68 ± 0.14^a C^
EPS R1	24	1.70 ± 0.11^a C^	1.37 ± 0.12^b B^	0.16 ± 0.10^b A^	0.26 ± 0.05^b A^	0.73 ± 0.25^a C^	4.22 ± 0.09^a B^	1.25 ± 0.03^a B^	5.40 ± 0.54^b C^	14.03 ± 7.32^a B^	9.01 ± 0.09^b B^
	48	1.40 ± 0.22^a B^	0.82 ± 0.12^a B^	0.49 ± 0.09^c A^	0.15 ± 0.01^a A^	1.92 ± 0.12^c B^	4.81 ± 0.37^b B^	1.70 ± 0.08^b B^	5.64 ± 0.55^b B^	2.86 ± 0.12^a B^	9.17 ± 0.09^b D^
	72	2.08 ± 0.10^b B^	1.44 ± 0.16^b C^	0.12 ± 0.09^b A^	0.26 ± 0.00^b B^	1.35 ± 0.18^b B^	5.25 ± 0.09^b B^	1.45 ± 0.11^a B^	5.62 ± 0.62^b B^	25.26 ± 19.65^a^	9.15 ± 0.13^b B^
	144	3.13 ± 0.18^c B^	1.70 ± 0.09^c B^	0.02 ± 0.01^a A^	0.53 ± 0.02^c A^	1.27 ± 0.26^b B^	6.65 ± 0.34^c B^	1.85 ± 0.20^b B^	3.21 ± 0.29^a A^	>100^b B^	6.73 ± 0.21^a A^
WCS	24	0.21 ± 0.05^a A^	0.26 ± 0.03^a A^	-	0.11 ± 0.07^a A^	0.09 ± 0.03^a A^	0.72 ± 0.12^a A^	0.79 ± 0.12^A^	2.33 ± 0.43 ^B^	ND	8.36 ± 0.15^c A^
	48	0.27 ± 0.05^a A^	0.34 ± 0.03^b A^	-	0.21 ± 0.01^b A^	0.17 ± 0.03^b A^	1.01 ± 0.05^a A^	0.79 ± 0.10^A^	1.66 ± 0.21^A^	ND	8.10 ± 0.06^c A^
	72	0.35 ± 0.06^a A^	0.36 ± 0.00^b A^	-	0.20 ± 0.03^b A^	0.17 ± 0.04^b A^	1.08 ± 0.02^a A^	0.99 ± 0.16^A^	1.83 ± 0.29 ^A^	ND	7.84 ± 0.11^b A^
	144	0.66 ± 0.22^b A^	0.51 ± 0.05^c A^	-	0.23 ± 0.05^b A^	0.26 ± 0.03^c A^	1.68 ± 0.35^b A^	1.28 ± 0.30^A^	2.30 ± 0.36 ^A^	ND	6.61 ± 0.19^a A^

Metabolite concentrations are expressed in mM, bacterial counts are expressed in ufc mL^−1^ Columns for the same carbon source with different lower case letter superscripts indicate significant differences along time (*p* > 0.05). Different capital letters superscripts indicate significant differences among carbon sources at the same sampling point (*p* < 0.05). * Sum of acetic, propionic, lactic, succinic, formic and pyruvic acids. ^+^ Counts at 0 h were 7.02 ± 0.08 log ufc mL^−1^. *WCS* without carbon source. -, under the detection limit, *ND* Not determined

The production of fermentation end-products from the catabolism of carbohydrates by *B. fragilis* was notably higher in glucose than in EPS and also in medium with the different EPS than in the medium without carbohydrates added all along the incubation period considered in the study (*p* < 0.05) (Table 1). Glucose was depleted in the culture medium after 24 h (data not shown) and metabolite levels remained without noticeable variations until 144 h of incubation. In contrast, total metabolites produced in medium with both EPS increased continuously until the end of fermentation, reaching clearly higher levels at the end of the incubation period in the culture with EPS E44 than in EPS R1 (*p* < 0.05). We analysed more in depth the evolution pattern of the different organic acids and SCFA formed during incubation (Table 1). The acetic to propionic acids ratio displayed higher values in glucose than in both EPS (*p* < 0.05) whereas, conversely, the propionic to succinic as well as the acetic to lactic acid ratios showed a trend (*p* < 0.1) to display higher values in cultures with EPS than with glucose. Regarding levels of the different microbial metabolites in cultures with EPS, the acetic, propionic and succinic acids increased until 24 h, decreased from 24 to 48–72 h, and increased again until the end of incubation. On the opposite, the concentration of lactic acid reached the maximum value at 48 h and then experienced a continuous decrease until the end of incubation (*p* < 0.05). As a consequence, at 48 h the acetic to lactic acid ratio reached its minimum value whereas the acetic to propionic ratio attained its maximum in cultures with EPS E44.

**Fig. 1:**
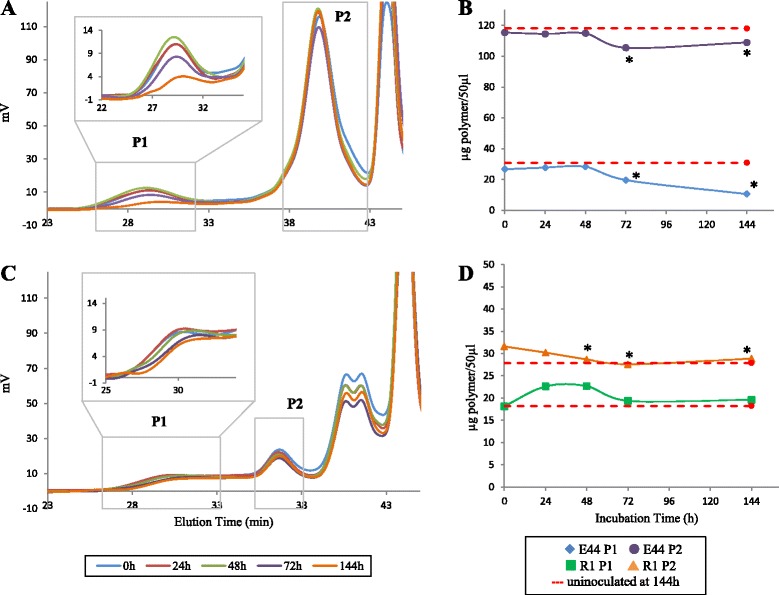
Time course of the bifidobacterial EPS degradation by *B. fragilis*. Refractive index chromatogram for degradation of EPS E44 peaks along time (**a**). Evolution of the total amount of EPS E44 peaks along time (**b**). Refractive index chromatogram for degradation of EPS R1 peaks along time (**c**). Evolution of the total amount of the two biggest peaks of EPS R1 fraction along time (**d**). Significant reduction of the amount of EPS in the corresponding peak with respect to time 0 is indicated by asterisks (*p* < 0.05). Dashed red lines indicate the amount of polymer for each peak after 144 h of incubation in the absence of *B. fragilis*

Since the only carbon source available in our experimental conditions were either glucose or EPS, we examined the evolution of the EPS fractions over time, looking for detectable variations in the amount and/or physicochemical characteristics of the polymer peaks that could demonstrate their consumption. Peaks of EPS E44, purified from the cellular biomass of *Bif. longum* E44, were according to that previously reported [22] and consisted in two polymers, one of 5 × 10^6^ Da (E44 P1) representing 18.8 % of the total mass, and a second one of 9 × 10^3^ Da (E44 P2), representing 81.2 % (Additional file 2: Figure S2). We ruled out any change in the molar mass distribution of the EPS E44 fraction along incubation in uninoculated MM (data not shown). Interestingly, the incubation of EPS with *B. fragilis* in the MM resulted in a significant decrease in the amount of the higher molar mass peak (E44 P1) of E44 from 48 h to 144 h of incubation (*p* < 0.05) (Fig. 1a and b) and of the smaller molar mass peak (E44 P2) between 48 and 72 h of incubation, remaining stable from 72 to 144 h (*p* < 0.05) (Fig. 1a and b). Molar mass distribution of EPS R1 was also in accordance with that previously reported by us [24], and consisted of three peaks; one of 1 × 10^6^ Da (R1 P1), another one of 2.35 × 10^4^ Da (R2 P2) and a small one of 4 × 10^3^ Da (R3 P3). This last peak overlapped in our analysis with an UV absorption peak and was then excluded from our study (Additional file 2: Figure S2). The total amount of the higher molar mass R1 polymer (R1 P1) did not show a decrease at the end of incubation, independently on the presence of *B. fragilis* (Fig. 1d). The amount of the medium size polymer (R1 P2) suffered an apparent depletion along incubation, which reached statistical significance at late incubation times (48, 72 and 144 h, *p* <0.05); however, the final amount of this polymer in the MM without *B. fragilis* was not significantly different (*p* > 0.05) from the values obtained in the culture with bacteria added (Fig. 1c and d) (statistical data not shown).

## Discussion

The use of bacterial EPS by *Bacteroides thetaiotaomicron* has been described before with HoPS produced by some lactic acid bacteria and *Streptococcus* spp. [6, 16, 25]. Although the use of HePS from bifidobacteria had not been definitely demonstrated yet, we have previously identified changes in the metabolism of *B. fragilis* in the presence of these polymers in an undefined medium [18]. Results from the current study demonstrated an effective growth of this specie in the presence of HePS. As compared to glucose, *Bacteroides* population levels attained with our EPS at early stages of growth were lower, but probably the slow utilization of these polymers and hence their availability as carbon source along incubation contributed to maintain the microbial levels at late states of growth, this phenomenon being more pronounced with EPS E44 than with EPS R1. Besides, a differential metabolic activity of *B. fragilis* in the presence of EPS as compared to glucose was evidenced. *B. fragilis* remained metabolically active in cultures with EPS for a longer period of time, with the highest activity corresponding to the cultures with the polymer E44. Variations obtained in metabolic profiles of *Bacteroides* cultures as depending on the carbon sources were similar to those indicated previously by us using an undefined medium [18]. Additionally, higher propionic to succinic acid ratios with complex carbon sources relative to glucose, similar to that found in the current study, have been previously reported [26].

In the present study we observed a clear shift in the metabolite production by *B. fragilis* during the time course of fermentation in the presence of EPS with respect to glucose, which was more pronounced with the polymer E44 than with R1. Coinciding with the depletion in the amount of EPS polymers from 48 h of incubation, a gradual increase in the concentrations of acetic, propionic and succinic acids and a decrease in the levels of lactic acid occurred until the end of incubation. It has been previously suggested that, in *Bacteroides,* the production of propionate through the succinate/propionate pathway could be a cell response to optimize cell energy production while keeping the intracellular redox balance [18, 19]. Although *B. fragilis* has the capacity to metabolize moderate amounts of lactic acid [27] as well as amino acids [28], our work was performed in a minimal medium, so that no amino acid sources were available and the scarce consumption of lactic acid that may occur does not explain the growth and metabolic activity of the bacterium in such conditions.

The results from SEC-MALS chromatography indicated that *B. fragilis* was able to use the EPS E44 produced by *Bif. longum* as a fermentable substrate. The chemical structure of EPS E44 has not been elucidated yet but it is known that EPS E44 contains glucose and galactose in its composition [22]. It is then possible that the saccharolytic enzymatic machinery of *B. fragilis* could include enzymes able to participate in the degradation of this complex substrate. On the other hand, changes in growth and metabolic patterns occurring at late stages of growth in EPS E44 could be related with the cessation of consumption of the smaller peak beyond 72 h of incubation, and hence with a scarcity of carbon source available from this time. Our results are not conclusive about the possible degradation of the EPS R1 fraction by *B. fragilis.* Even though there were no significant changes attributable to the activity of *B. fragilis* in the EPS peaks of high and medium molar mass, we could not rule out changes in the amount of the smallest polymer, not considered in our study because the overlapping with a protein peak. Variations in this small polymer may provide a possible explanation for the increased metabolic activity evidenced in cultures of *B. fragilis* until 72 h of incubation in the presence of the EPS R1 fraction as compared to the control in MM without carbon sources added. The EPS R1 fraction is formed by glucose, galactose and rhamnose [24] and only the chemical structure of the high molar mass peak has been determined to date, which is composed by 50 % rhamnose [29]. Although we know that the presence of both EPS stimulates the production of α-glucosidase by *B. fragilis*, the high molar mass polymer of the EPS R1 fraction lacks the α-linkages targeted by this enzymatic activity [29]. This together with the inability of *B. fragilis* to ferment rhamnose [28], could pose difficulties for the use as fermentable substrate of the EPS R1 by this microorganism.

The ability of *B. fragilis* to use bifidobacterial EPS may provide this microorganism with a long-term available complex carbon source, thus enhancing its survival and conferring it a selective advantage in environments where nutrients are scarce, such as the case of the human large intestine. *Bacteroides* plays an important role in the utilization of indigestible dietary compounds and complex polymers secreted by other microorganisms [6]. Some EPS and capsular polysaccharides are involved in adhesion to eukaryotic cells, biofilm formation and protection of several species against the gastrointestinal stressing factors [14]. In this way the ability of *Bacteroides* to degrade these polymers may confer this microorganism a role in the regulation of microbial relationships in the gut ecosystem. Fermentation of EPS in faecal cultures lead to an increase in propionic acid production [17], most likely due to the metabolic activity of members from the genus *Bacteroides*.

## Conclusions

The present work is the first report demonstrating that *B. fragilis* is able to use some EPS produced by a bifidobacteria as substrate for growth, which resulted in a partial polymer consumption and the concomitant release of metabolic end products from its fermentation. By extrapolating these findings to the human gut, it may be hypothesized that the feeding relationship between microbial EPS and *Bacteroides* could have an impact in the SCFA production balance in the gut, which is ultimately related with the human health.

## Abbreviations

EPS, Exopolysaccharide; GAMc, Gifu Anaerobic Medium with cysteine; HePS, Heteroexopolysaccharides; HoPS, Homopolysaccharides; MM, Minimal medium; SCFA, Short Chain Fatty Acids; SEC-MALLS, Size-Exclusion chromatography-Multiangle Laser Light Scattering

## Additional files

Additional file 1: Figure S1. Thin layer chromatography of the digestion of glycogen and EPS E44 and R1 fractions with enzymes α-amylase, pullulanase, and with a mixture of α-amylase and pullulanase. (PPTX 522 kb) Additional file 2: Figure S2. Size exclusion chromatography (SEC-MALS) analysis of the EPS E44 (A) and EPS R1 (B) fractions purified from the cell biomass of *Bifidobacterium longum* E44 and *Bifidobacterium animalis* subsp. *lactis* R1, respectively. Refractive index detector (blue line) for detection and quantification of EPS peaks, PDA detector (green line) set at 280 nm to identify the presence of proteins, and the multiangle laser light scattering (MALS) for molar mass distribution of the EPS fractions (red line). (PPTX 758 kb)
