# Supplementary material for: Bacteroides fragilis metabolises exopolysaccharides produced by bifidobacteria
Source: BMC Microbiol. 2016 Jul 15;16:150. doi: 10.1186/s12866-016-0773-9 (PMC4946188; doi:10.1186/s12866-016-0773-9)
Supplement: Additional file 1: Figure S1. — Thin layer chromatography of the digestion of glycogen and EPS E44 and R1 fractions with enzymes α-amylase, pullulanase, and with a mixture of α-amylase and pullulanase. (PPTX 522 kb) [file 12866_2016_773_MOESM1_ESM.pptx]

## Slide 1
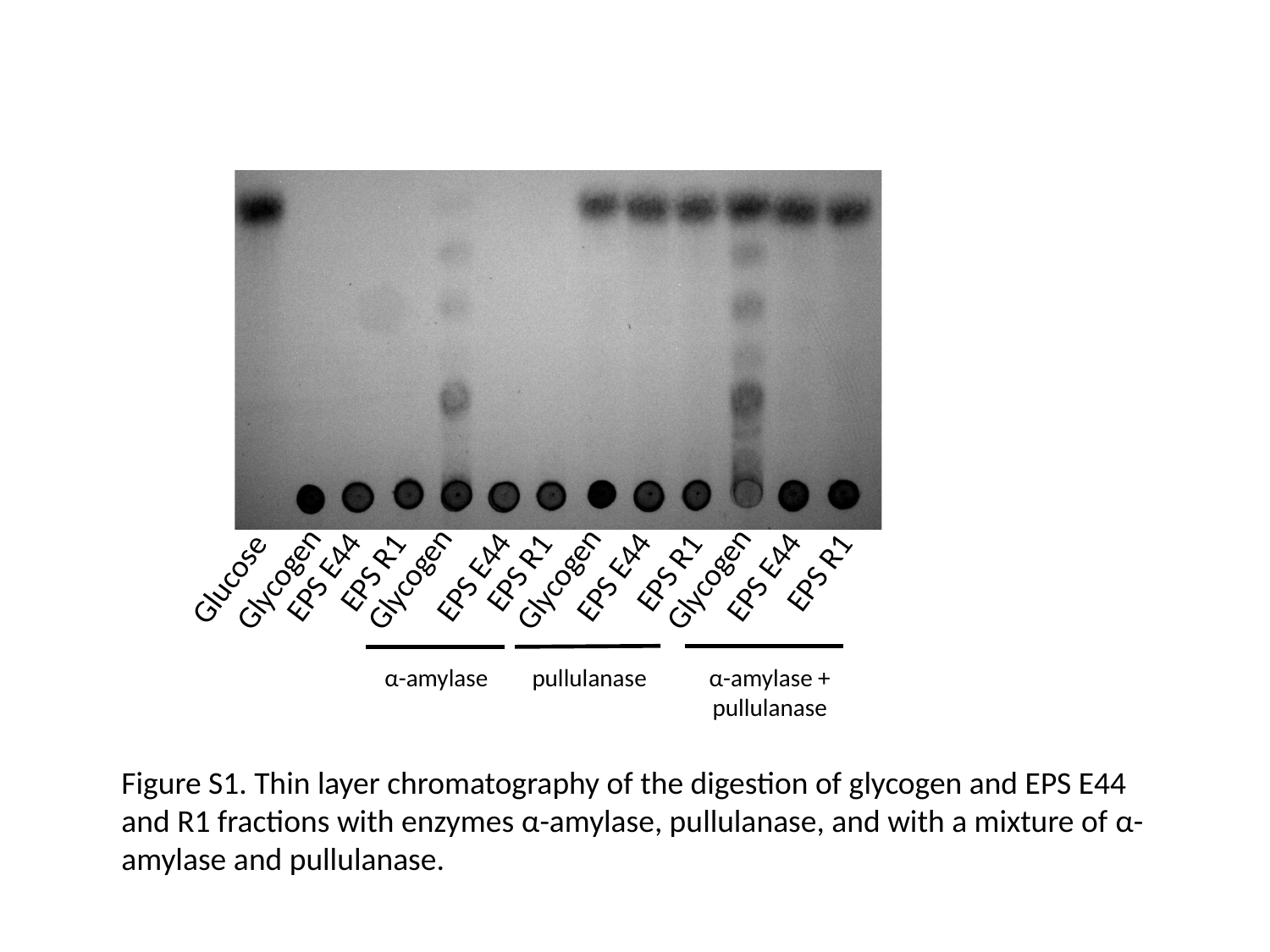

EPS R1
EPS R1
EPS R1
EPS R1
EPS E44
EPS E44
EPS E44
EPS E44
Glucose
Glycogen
Glycogen
Glycogen
Glycogen
α-amylase
pullulanase
α-amylase + pullulanase
Figure S1. Thin layer chromatography of the digestion of glycogen and EPS E44 and R1 fractions with enzymes α-amylase, pullulanase, and with a mixture of α-amylase and pullulanase.
