# Supplementary material for: Bacteroides fragilis metabolises exopolysaccharides produced by bifidobacteria
Source: BMC Microbiol. 2016 Jul 15;16:150. doi: 10.1186/s12866-016-0773-9 (PMC4946188; doi:10.1186/s12866-016-0773-9)
Supplement: Additional file 2: Figure S2. — Size exclusion chromatography (SEC-MALS) analysis of the EPS E44 (A) and EPS R1 (B) fractions purified from the cell biomass of Bifidobacterium longum E44 and Bifidobacterium animalis subsp. lactis R1, respectively. Refractive index detector (blue line) for detection and quantification of EPS peaks, PDA detector (green line) set at 280 nm to identify the presence of proteins, and the multiangle laser light scattering (MALS) for molar mass distribution of the EPS fractions (red line). (PPTX 758 kb) [file 12866_2016_773_MOESM2_ESM.pptx]

## Slide 1
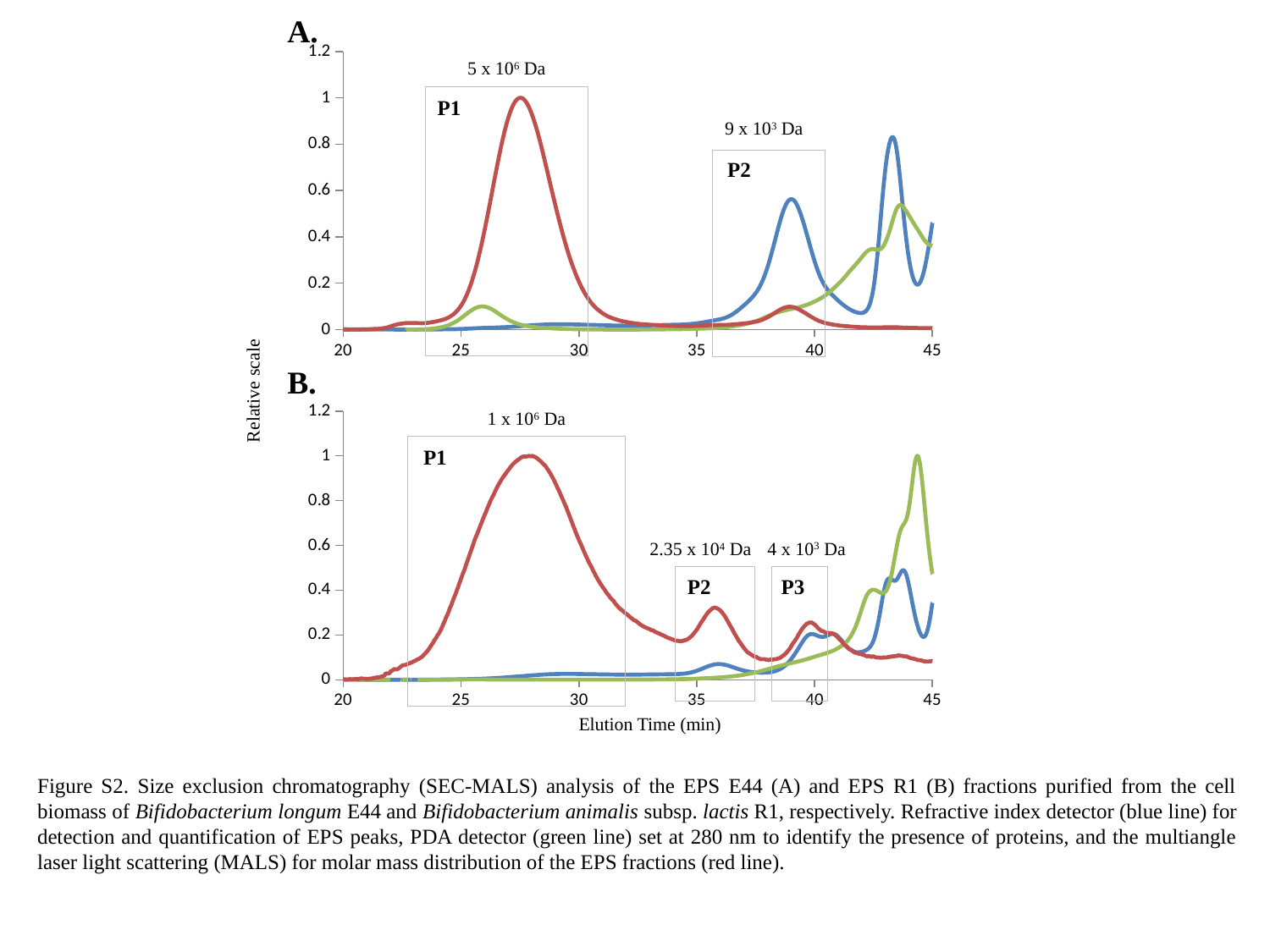

A.
### Chart
| Category | | | |
|---|---|---|---|5 x 10^6^ Da
P1
9 x 10^3^ Da
P2
B.
Relative scale
### Chart
| Category | | | |
|---|---|---|---|1 x 10^6^ Da
P1
4 x 10^3^ Da
2.35 x 10^4^ Da
P2
P3
Elution Time (min)
Figure S2. Size exclusion chromatography (SEC-MALS) analysis of the EPS E44 (A) and EPS R1 (B) fractions purified from the cell biomass of Bifidobacterium longum E44 and Bifidobacterium animalis subsp. lactis R1, respectively. Refractive index detector (blue line) for detection and quantification of EPS peaks, PDA detector (green line) set at 280 nm to identify the presence of proteins, and the multiangle laser light scattering (MALS) for molar mass distribution of the EPS fractions (red line).
